# Breast cancer assessment under neoadjuvant systemic therapy using thoracic photon-counting detector computed tomography in prone position: a pilot study

**DOI:** 10.1186/s41747-025-00576-z

**Published:** 2025-03-28

**Authors:** Claudia Neubauer, Johanna Nattenmüller, Fabian Bamberg, Marisa Windfuhr-Blum, Jakob Neubauer

**Affiliations:** Department of Diagnostic and Interventional Radiology, Medical Center–University of Freiburg, Faculty of Medicine, University of Freiburg, Freiburg im Breisgau, Germany

**Keywords:** Breast neoplasms, Neoadjuvant therapy, Neoplasm (residual), Prone position, Tomography (x-ray computed)

## Abstract

**Background:**

Accurate assessment of treatment response to neoadjuvant systemic therapy (NAST) in breast cancer is important prior to surgery. We aimed at evaluating the feasibility of thoracic photon-counting detector computed tomography (PCCT) in assessing treatment response in breast cancers following NAST.

**Methods:**

We retrospectively included patients with newly diagnosed breast cancer who received contrast-enhanced thoracic PCCT in prone position before and after NAST. Three experienced radiologists measured tumor size, tumor area, iodine uptake within tumors, number of suspicious breast lesions and of suspicious axillary lymph nodes before and after NAST. We compared the initial tumor size to contrast-enhanced magnetic resonance imaging (MRI), the residual tumor size after NAST to histopathology.

**Results:**

Eighteen PCCT exams in nine patients aged 58 ± 14 years (mean ± standard deviation) were analyzed. After NAST, PCCT correctly identified a reduction in tumor burden in 9 of 9 cases and a complete response in 2 of 2 cases, with a significant reduction in tumor size, area, T-stage, number of suspicious breast lesions and of suspicious lymph nodes (*p* < 0.001 for all) as well as reduction in cutaneous infiltration (*p* = 0.010). Mean and maximum iodine uptake showed a nonsignificant reduction in cases with residual tumor after NAST (*p* = 0.092 and 0.363).

**Conclusion:**

These preliminary findings suggest that thoracic PCCT can accurately detect local changes in breast cancer after NAST.

**Relevance statement:**

Thoracic PCCT offers promising potential for accurately assessing breast cancer response to NAST.

**Trial registration:**

German Clinical Trials Register DRKS00028997.

**Key Points:**

- Prone thoracic contrast-enhanced photon-counting detector computed tomography (PCCT) can accurately detect reductions in tumor size, area, and T-stage.
- Prone PCCT can identify a decrease in the number of suspicious axillary lymph nodes.
- This technique shows promising results in identifying breast cancer response to neoadjuvant systemic therapy (NAST).

**Graphical Abstract:**

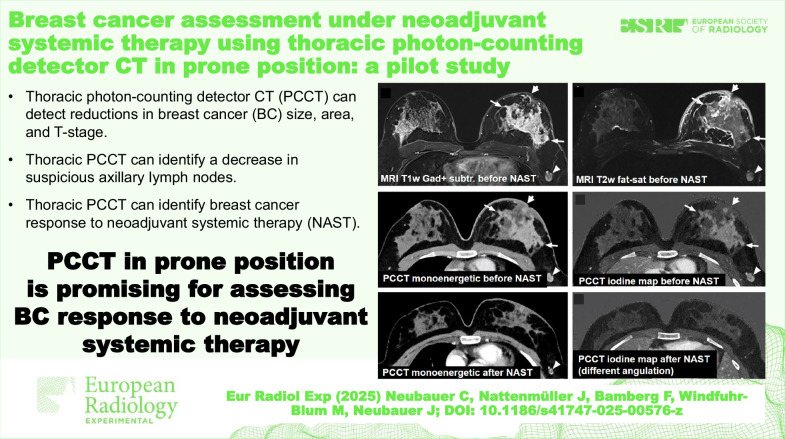

## Background

Neoadjuvant systemic therapy (NAST) is widely employed to reduce breast cancer size prior to surgery. However, not all patients respond to NAST with a reduction in tumor size or achieve a complete response. Pathological complete response, which is associated with improved event-free and recurrence-free survival, is attained in only about 28–35% of cases following NAST [1, 2]. Therefore, accurately assessing the response or nonresponse to NAST before surgery or other subsequent treatment is crucial.

Among the various imaging techniques available, magnetic resonance imaging (MRI), along with sonography [3], is particularly noted for its ability to accurately measure and assess tumor size and tumor volume after NAST [4–6]. Additionally, MRI can evaluate tumor cellularity using diffusion-weighted imaging [7, 8] and functional tumor volume using dynamic contrast-enhanced sequences [9]. Positron emission tomography also plays a role in detecting metabolic activity in tumors before and after NAST [10].

An alternative to breast MRI is dedicated breast computed tomography (CT), a specialized CT equipment designed for sequential examination of the two breasts, although it does not assess the axillary regions. Dedicated breast CT has shown potential in monitoring tumor volume changes after NAST [11] and in predicting pathologic outcomes [12]. In fact, for detecting residual tumor, especially ductal carcinoma *in situ*, calcifications or luminal subtypes, dedicated contrast-enhanced breast CT has even demonstrated greater accuracy than breast MRI, with preoperative prediction of pathologic complete response comparable to that of breast MRI [5, 13]. However, dedicated breast CT is mainly performed in research settings and is not yet widely accessible.

Photon-counting detector CT (PCCT) is a promising new CT technology [14] that offers higher spatial resolution than conventional CT and enables additional spectral analysis of iodine uptake. Thoracoabdominal contrast-enhanced staging CT is commonly used in breast cancer patients, and imaging of both breasts is included without the need for additional radiation, contrast agent, or examination time. At our hospital, we perform PCCT in prone position, similar to breast MRI, for breast cancer patients requiring thoracic PCCT. This prone-positioned contrast-enhanced thoracic PCCT has already proved to surpass digital mammography in locoregional breast cancer assessment, with significant improvements in diagnostic accuracy for T-classification, number, and distribution of tumor masses [15, 16]. Unlike mammography, its diagnostic performance is not affected by breast density [15, 16]. Moreover, thoracic PCCT allows for the simultaneous visualization of axillary lymph nodes. Although no absolute accurate restaging of nodal status after NAST exists [17], at least radiomics from both dedicated breast CT and thoracic CT have shown promising diagnostic performance for predicting residual axillary nodal metastasis after NAST [18, 19], comparable to sonography and MRI [20].

To our knowledge, no studies have yet investigated the response to NAST using thoracic PCCT.

The aim of this study was to evaluate the diagnostic performance of thoracic contrast-enhanced PCCT in prone position for assessing response of breast tumors and axillary lymph node metastasis to NAST.

## Methods

### Study design and participants

Our retrospective pilot cohort study was conducted at our university medical center. We retrospectively identified all female patients with breast cancer who underwent a prone-positioned contrast-enhanced PCCT between December 2021 and August 2023. Eligible participants included all women newly diagnosed with breast cancer who received NAST and had thoracic or thoracoabdominal PCCT scans before NAST and after completing NAST but prior to surgery. Supplementary breast MRI scans performed before NAST were welcomed but not mandatory for inclusion in this study. Histopathological analysis of formalin-embedded tumor specimens was performed at our local university histopathological institute. Tumor sizes were received from histopathological reports of specimens after tumor surgery post-NAST and served as reference standard after treatment. Written informed consent was obtained from all participants.

The study was conducted in accordance with the Declaration of Helsinki and received approval by the local ethics committee.

### Imaging protocols

#### PCCT

All participants underwent contrast-enhanced thoracic CT investigations at our PCCT scanner with dual source technology (NAEOTOM Alpha, Siemens Healthineers, Erlangen, Germany) in prone position. The first PCCT was always performed in the context of thoracoabdominal staging after newly diagnosed breast cancer and before NAST. The performance of a thoracic PCCT after NAST is no routine procedure. Therefore, the number of finally included patients is small. Clinical indications for further follow-up PCCT after NAST and before surgery had several different individually determined reasons, as shown in Table 1. Prone positioning was aligned with the positioning used in breast MRI with both breasts hanging freely between two pillows, as described in detail before, to allow artifact-free PCCT imaging in standard prone breast imaging position [15]. Helical acquisition was performed after body weight adapted bolus injection of iodinated contrast agent (80 mL + 0.5 mL/kg body weight of iopromide 370 mg/mL up to a maximum of 140 mL; Bayer Healthcare, Leverkusen, Germany) and a fixed delay of 85 s, followed by a saline chaser with a flow of 3 mL/s via a 20-gauge catheter.

**Table 1:** Patient characteristics and therapy

	Number	%
Number of patients enrolled	9	100
Age, years (mean ± standard deviation)	58.1 ± 14.4	
Menopausal status		
Premenopausal	2	22.2
Postmenopausal	7	77.8
Neoadjuvant systemic therapy		
4× Epirubicin/Cyclophosphamide → Paclitaxel/Carboplatin	1	11.1
4× Epirubicin/Cyclophosphamide → Paclitaxel/Pertuzumab/Trastuzumab	4	44.4
4× Epirubicin/Cyclophosphamide → Paclitaxel/Carboplatin/Pembrolizumab	1	11.1
Paclitaxel/Carboplatin/Pembrolizumab → 4× Epirubicin/Cyclophosphamide	1	11.1
Paclitaxel → 4× Epirubicin/Cyclophosphamide	1	11.1
Palbociclib + Letrozol	1	11.1
Indication for second thoracic photon-counting computed tomography		
Control of pulmonary findings	6	66.7
Control of suspicious intrathoracic lymph nodes	1	11.1
Sepsis with pneumonia	1	11.1
Aortic aneurysm	1	11.1
Surgery after neoadjuvant systemic therapy		
Breast-conserving surgery	3	33.3
Mastectomy	6	66.7
Tumor stage after surgery		
ypT0	2	22.2
ypTis	2	22.2
ypT1a	2	22.2
ypT1b	1	11.1
ypT1c	1	11.1
ypT2	1	11.1

The tube voltage of PCCT was set to 120 kVp, associated with a quality reference of 142 mAs. In addition to conventional thoracic reconstructions, we performed transversal reconstructions over both breasts, the anterior thoracic wall and both axillary regions, including a monoenergetic 65-keV reconstruction, an iodine map, and a virtual unenhanced reconstruction. These special reconstructions covered a field of view of 34 cm, a matrix of 1,024 × 1,024 pixels, a slice thickness of 2 mm, a slice increment of 2 mm, and kernel Br40 with iterative reconstruction strength 3. Since breast imaging was performed implicitly in the setting of clinically indicated thoracic PCCT without the application of additional radiation or contrast agents, no dose measurements were conducted.

#### MRI

Of nine participants, eight underwent additional clinically indicated standard multiparametric 1.5-T (2/8 participants, MAGNETOM Avanto and MAGNETOM Aera, Siemens Healthineers, Erlangen, Germany) or 3-T breast MRI (6/8 participants, MAGNETOM Vida, Siemens Healthineers, Erlangen, Germany), with an 18-channel breast coil. The following sequences were performed: transversal fat-saturated and non-fat-saturated T2-weighted (1.5-T MRI), transversal T2-weighted turbo spin-echo Dixon (3-T MRI), and T1-weighted sequences prior to and dynamically after application of 0.1 mmol/kg of gadoteridol (ProHance, Bracco, Konstanz, Germany) with creation of subtraction images. At 3-T MRI, additionally ultrafast TWIST imaging was performed after contrast agent application. Diffusion-weighted imaging was included in 6/8 MRI examinations. All MRI examinations evaluated in this study were performed prior to NAST.

### Image analysis

Pre- and post-NAST PCCT examinations were pseudonymized and randomly assigned into two groups for evaluation. Evaluation was performed by three experienced radiologists (Reader 1, 12 years CT imaging and 6 years breast imaging; Reader 2, 13 years CT imaging and 5 years breast imaging; and Reader 3, 14 years CT imaging and 6 months breast imaging). All readers were blinded to patient data and treatment. They independently evaluated the pseudonymized PCCT examinations of the first group and after a time gap of at least 5 weeks to ensure disengagement from the previous evaluations of the second group. The first group consisted of one randomly assigned pre- or post-neoadjuvant examination of each of the 9 participants in random order, the second group consisted of the corresponding post- or pre-neoadjuvant examination of each patient which were again presented in a random order.

Readers were informed about the side of breast malignancy. Measurements performed on transversal slides included largest tumor diameter (in mm), largest tumor area (in mm^2^), number of suspicious lesions, focality, visual presence of infiltration of dermis, pectoralis muscle and/or thoracic wall, and the presence of suspicious axillary lymph nodes. In case of suspicious lymph nodes, the short axis diameter of the biggest suspicious axillary lymph node was measured (in mm). Lymph nodes were regarded as suspicious, if they met one or more of the following criteria: marked asymmetry, cortical thickening, loss of the fatty hilum, round shape, irregular margin, heterogeneous cortex, and/or surrounding edema, whereas they were considered as being normal when they were symmetric with homogeneous enhancement, thin cortex and preserved fatty hilum.

The maximum and mean absorption of tumors and aorta were assessed in the monoenergetic reconstruction, the iodine map and the virtual unenhanced reconstruction (in HU using small ROIs). Iodine quantity was calculated from the mean and maximum HU measurements of the tumors as measured by reader 1 in the iodine map to receive an iodine value in mg/mL.

After a gap of 6 weeks to ensure readers were disengaged from the previous evaluations, tumor size was additionally assessed by reader 1 and 2 in breast MRI before NAST as far as available (8/9 patients). Furthermore, as there has been a discrepancy in tumor size as measured in PCCT after NAST compared to histopathology in one participant with multicentric diffuse breast cancer and another with multicentric disease, 2 readers were asked to measure again the largest residual tumor expansion in those two pseudonymized cases in PCCT. These measurements were included in the comparison of tumor size to histologically residual vital tumor size. Information about the residual size of vital tumor after NAST was withdrawn from the histopathological report after tumor surgery. Pathological complete response was defined as the absence of invasive or *in situ* residuals in the breast, while radiological complete response was defined as the absence of distinguishable tumor residuals in PCCT.

Image quality and certainty of diagnosis were evaluated by each reader in PCCT examinations before and after NAST with the 4-point Likert scale (1 = very high, 2 = high, 3 = moderate, 4 = low/not sufficient for diagnosis).

All investigations were evaluated with our standard viewing software (DeepUnity Diagnost, Dedalus, Bonn, Germany) using calibrated diagnostic monitors under standard diagnostic conditions.

### Statistics

Tumor size at PCCT was compared with reference measurements at breast MRI before NAST and with histopathology after NAST using Pearson’s *r* correlation. Spearman’s ρ test was used to compare T-stage at PCCT with MRI before NAST and with histopathology after NAST. Wilcoxon signed rank test was used to compare tumor size, area, T-stage, density, iodine uptake, focality, lymph node number, image quality and certainty of findings before and after NAST for each reader as well as averaged for all readers. Agreement for tumor diameter measurements between PCCT and breast MRI as reference standard before NAST as well as between PCCT and histopathology as reference standard after NAST was assessed by Bland-Altman plots.

Inter-rater reliability was calculated using intraclass correlation coefficient for main tumor size and area, number of suspicious lesions, measurements of mean tumor density, and iodine uptake, with values < 0.50 indicating poor reliability, ≥ 0.50 but < 0.75 moderate reliability, ≥ 0.75 but < 0.90 good reliability, and ≥ 0.90 excellent reliability.

Fleiss *κ* analysis was used for evaluating inter-rater reliability for focality, cutis and wall infiltration, and number of suspicious axillary lymph nodes; *κ* values of 0.00 were interpreted to indicate no agreement; *κ* ≤ 0.20 slight agreement; 0.20 < *κ* ≤ 0.40 fair agreement; 0.40 < *κ* ≤ 0.60 moderate agreement; 0.60 < *κ* ≤ 0.80 substantial agreement, and 0.80 < *κ* ≤ 1.00 represented an almost perfect agreement.

Comparing inter-rater reliability before and after NAST was performed by using the Wilcoxon signed rank test.

For nominal and ordinal numbers, the median was calculated, for continuous values the mean was used and displayed in the tables.

A *p*-value < 0.05 was considered to indicate statistical significance. Statistical analyses were performed with R (Version 4.3.2).

## Results

### Patients’ population under NAST

**Fig. 1:**
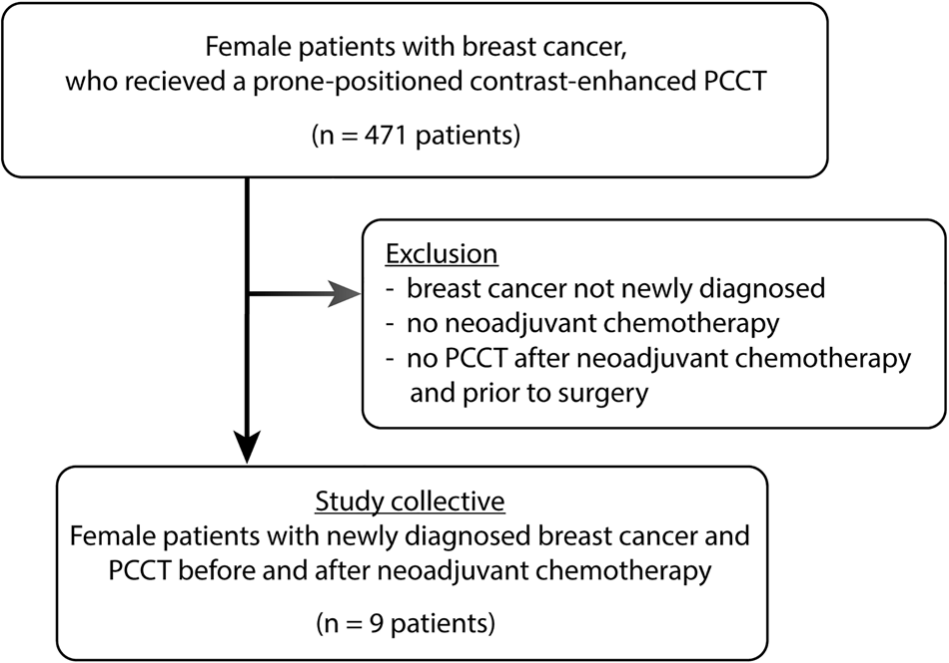
Flowchart showing patients investigated with photon-counting detector computed tomography, exclusion criteria and final study population. PCCT, Photon-counting detector computed tomography

From 471 female patients who received a contrast-enhanced PCCT in prone position, 9 patients, aged 58 ± 14 years (mean ± standard deviation), met our inclusion criteria and could be enrolled in our study (Fig. 1). Patient’s characteristics and therapy are summarized in Table 1, histopathological results from initial tumor biopsy are provided in Table 2. Primarily, a routine staging PCCT in prone positioning was performed after newly diagnosed breast cancer and before NAST. Only those patients with an indication for another clinically indicated and necessary prone-positioned PCCT after NAST and before surgery could be finally included in this study. Therefore, the number of finally included patients was small. Indications for a second PCCT after NAST included the control of suspicious pulmonary findings such as small solid or ground glass nodules (*n* = 6) or intrathoracic lymph nodes (*n* = 1), an assessment of a sepsis with pneumonia (*n* = 1), or the reevaluation of an aortic aneurysm (*n* = 1). Eight out of nine patients also received a breast MRI before NAST. All patients underwent breast surgery with a final histopathological report.

**Table 2:** Tumor characteristics including histopathology

	Number	%
Initial histopathology of cancer		
No special type	4	44.4
No special type + Ductal carcinoma *in situ*	4	44.4
Lobular type	1	11.1
Clinical T-stage		
cT1a	0	0
cT1b	0	0
cT1c	3	33.3
cT2	3	33.3
cT3	0	0
cT4	3	33.3
Tumor grade		
Grade 1	1	11.1
Grade 2	5	55.6
Grade 3	3	33.3
ER		
Negative	4	66.7
Positive	5	33.3
PR		
Negative	6	66.6
Positive	3	33.3
HER2		
Negative	6	66.6
Positive	3	33.3
Hormone and HER2 type		
ER+/PR+/HER2- (Luminal A)	3	33.3
ER+/PR-/HER2+ (Luminal B)	1	11.1
ER-/PR-/HER2+ (HER2 positive)	2	22.2
Triple-negative type	3	33.3
Proliferation index (Ki-67 / MiB-1)		
< 10%	1	11.1
> 10%	8	88.9

*ER* Estrogen receptor, *HER2* Human epidermal growth factor receptor, *PR* Progesterone receptor

### Tumor assessment before and after neoadjuvant systemic therapy

**Fig. 2:**
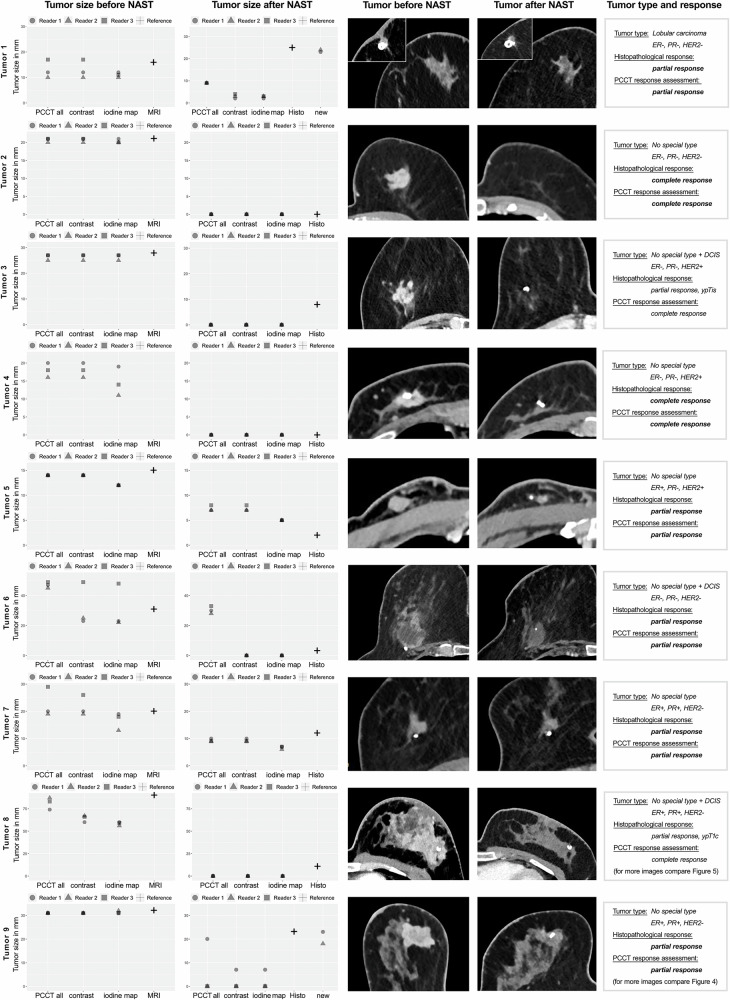
Comparison of tumor diameter measurements in thoracic contrast-enhanced photon-counting detector computed tomography with total (PCCT all) and enhancing (contrast) tumor measurements in the monoenergetic reconstruction and iodine map (iodine map) before neoadjuvant systemic treatment *versus* tumor diameter measurements in breast MRI as reference standard (left column, MRI measurement = cross) and after neoadjuvant systemic treatment *versus* histopathology (Histo) as reference (right column, histopathology = cross) for each reader. Tumor 1 was diffuse with measurement of a representative area in MRI as well as PCCT before and after NAST; here and for tumor 9, additionally after NAST 2 readers measured again tumor spread in the PCCT (new). There was no MRI available for assessment of tumor 4. Representative PCCT images of measured tumors before and after NAST, as well as individual tumor and response characteristics (right column), are shown for each tumor. DCIS, Ductal carcinoma *in situ*; ER, Estrogen receptor; HER2, Human epidermal growth factor receptor; MRI, Magnetic resonance imaging; NAST, Neoadjuvant systemic therapy; PCCT, Photon-counting detector computed tomography; PR, Progesterone receptor

**Fig. 3:**
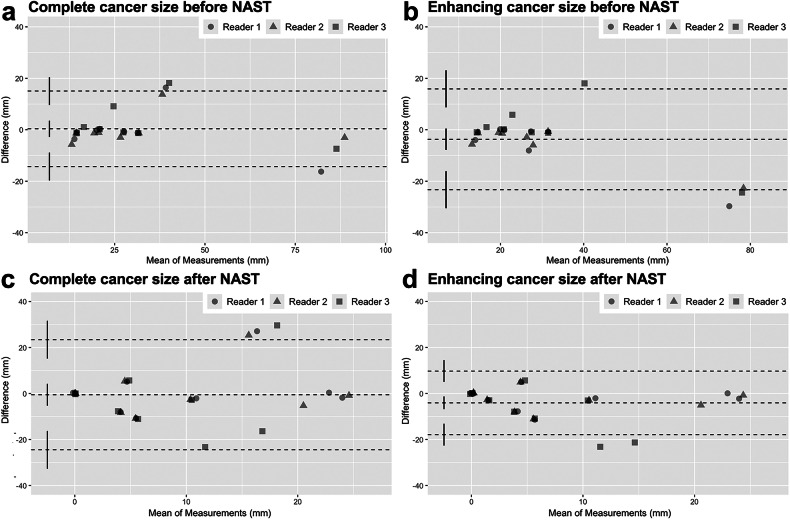
Agreement (Bland-Altman plots) between complete (**a**, **c**) and enhancing (**b**, **d**) tumor diameter measurements in thoracic contrast-enhanced photon-counting detector computed tomography before neoadjuvant systemic treatment *versus* tumor diameter measurements in breast MRI as reference standard (**a**, **b**) and after neoadjuvant systemic treatment *versus* histopathology as reference (**c**, **d**) for each reader. NAST, Neoadjuvant systemic therapy

Enhancing tumor size in initial PCCT showed a significant correlation to reference tumor size of initial breast MRI as assessed in the monoenergetic reconstructions (averaged *r* = 0.940, *p* < 0.001) and the iodine map (averaged *r* = 0.919, *p* < 0.001) (compare Figs. 2, 3b) as did the initial T-stage in PCCT compared to MRI before NAST (averaged ρ = 0.903, *p* < 0.001). A comparison of finally assessed enhancing tumor size measurements in PCCT to residual vital tumor size as provided by histopathology after tumor surgery, also showed a significant correlation for the two most experienced readers in breast imaging in the monoenergetic reconstructions (averaged *r* = 0.874, *p* = 0.002; for all readers with finally assessed values by reader 1 and 2 compare Figs. 2, 3d) and the iodine map (averaged *r* = 0.888, *p* < 0.001). Equally, T-stage assessed by PCCT correlated significantly to histopathological yT-stage after NAST (averaged all readers ρ = 0.786, *p* = 0.012).

For all readers, after NAST, a significant decline of largest enhancing tumor size could be perceived in the monoenergetic reconstructions and the iodine map (each averaged *p* < 0.001). The detailed tumor size measurements for each reader in PCCT before and after NAST, in MRI before NAST, and histopathology after NAST are provided in Supplementary Table S1.

The largest tumor area showed a significant reduction in the monoenergetic reconstructions and in the iodine map (each averaged *p* < 0.001). Accordingly, the local T-stage was significantly reduced from a median of 2 (IQR 3) before to a median of 0 (IQR 1, with 0 representing no measurable tumor T-stage due to no detectable residual tumor) after NAST for each reader (averaged *p* < 0.001). Cutis infiltration was recognized by all readers in 2/9 cases before NAST (case 1 and 8) and in 0/9 cases after NAST with a significant reduction (averaged *p* = 0.010). Pectoralis infiltration was present in 1/9 cases, as described by 2 readers before NAST (case 6). However, due to the small number of cases and the fact that one reader still identified an infiltration after NAST, the reduction was not statistically significant (averaged *p* = 0.500). Number of additional suspicious lesions in the breast showed a marked reduction (averaged *p* < 0.001).

**Fig. 4:**
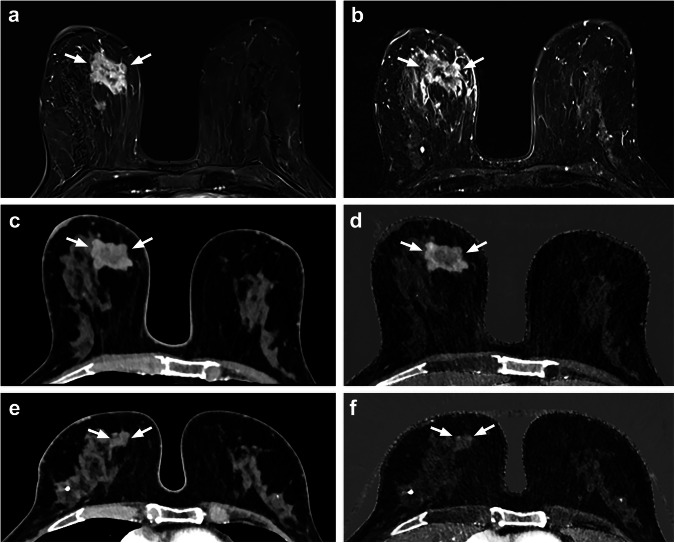
Patient with right-sided breast tumor (arrow) of no special type, G2, ER+/PR+/HER2-, visualized in breast MRI at initial diagnosis with T1w-subtraction after contrast administration (**a**) and fat-saturated T2w sequence (**b**). Photon-counting detector computed tomography with monoenergetic reconstruction (**c**, **e**) and iodine map (**d**, **f**) shows the tumor at initial tumor staging before NAST (**c**, **d**) and with a marked decline of tumor size after NAST (**e**, **f**). ER, Estrogen receptor; HER2, Human epidermal growth factor receptor; MRI, Magnetic resonance imaging; NAST, Neoadjuvant systemic therapy; PR, Progesterone receptor

**Fig. 5:**
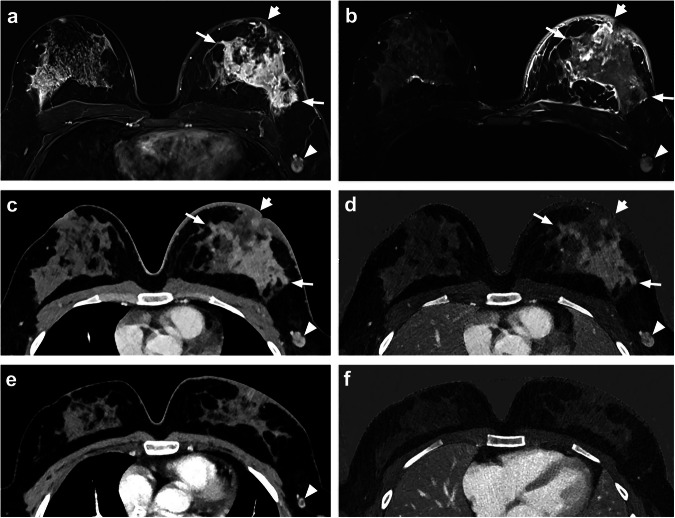
Patient with left-sided multicentric inflammatory breast tumor (long arrow) of no special type, G3, ER-/PR-/HER2+, with cutis infiltration (on the presented slices cutis thickening is indicated with a short wide arrow) and axillary lymph node metastasis (arrowhead) visualized in breast MRI at initial diagnosis with T1w-subtraction after contrast administration (**a**) and fat-saturated T2w sequence (**b**). Photon-counting detector computed tomography with monoenergetic reconstruction (**c**, **e**) and iodine map (**d**, **f**) shows the tumor before NAST (**c**, **d**) and with a marked decrease in tumor size and tumor enhancement to a point where it is no longer clearly delineated and a clear reduction in lymph node size following NAST (**e**, **f**; **f** with slightly different angulation, as only **e** could be angulated for visual presentation to show residual tumor and lymph node in one slice similar to pretherapeutic PCCT). ER, Estrogen receptor; HER2, Human epidermal growth factor receptor; NAST, Neoadjuvant systemic therapy; PCCT, Photon-counting detector computed tomography; PR, Progesterone receptor

The results are summarized in Tables 3 and 4 with averaged values and values for each reader. Examples of breast tumors in MRI and PCCT before NAST and in PCCT after NAST are shown in Figs. 4 and 5. Further data for number of suspicious lesions, cutis and pectoralis infiltration are provided in Supplementary Table S2.

**Table 3:** Comparison of tumor size and T-stage from photon-counting detector computed tomography measurements with MRI measurements before and with histopathology after neoadjuvant systemic therapy

		Before NAST: PCCT—MRI		After NAST: PCCT—histopathology	
		Correlation	*p*-value	Correlation	*p*-value
Tumor size	Monoenergetic reconstruction	*r* = 0.940 (0.978/0.988/0.899)	< 0.001 (< 0.001/< 0.001/0.002)	*r* = 0.874 (0.880/0.870)	0.002 (0.002/0.002)
	Iodine map	*r* = 0.919 (0.976/0.958/0.859)	< 0.001 (< 0.001/< 0.001/0.006)	*r* = 0.888 (0.894/0.885)	0.001 (0.001/0.002)
T-stage		ρ = 0.903 (0.898/0.933/0.932)	< 0.001 (0.002/0.001/0.001)	ρ = 0.752 (0.786*/*0.722)	< 0.001 (0.012/0.028)

*MRI* Magnetic resonance imaging, *NAST* Neoadjuvant systemic therapy, *PCCT* Photon-counting detector computed tomography

**Table 4:** Comparison of tumor characteristics and subjective evaluation of image quality and certainty of findings before and after neoadjuvant systemic therapy

		Before NAST	After NAST	*p*-value
Tumor size (mm)	Monoenergetic reconstruction	26.81 (SD 15.66)	2.44 (SD 3.62)	< 0.001 (0.005/0.005/0.002)
	Iodine map	24.63 (SD 14.88)	1.85 (SD 2.68)	< 0.001 (0.002/0.004/0.002)
Tumor area (mm^2^)	Monoenergetic reconstruction	374.50 (SD 468.40)	11.04 (SD 17.25)	< 0.001 (0.005/0.004/0.002)
	Iodine map	336.96 (434.72)	5.30 (SD 7.77)	< 0.001 (0.002/0.004/0.002)
T-stage (1–4, 0 = no residual tumor)		2 (IQR 3)	0 (IQR 1)	< 0.001 (0.011/0.011/0.011)
Additional suspicious lesions (number)		4 (IQR 4)	1 (IQR 3)	< 0.001 (0.099/0.028/0.018)
Suspicious lymph nodes (number)		1.56 (SD 2.24)	0.04 (SD 0.19)	< 0.001
Image quality (Likert scale)		1 (IQR 0)	1 (IQR 0)	
Certainty of findings (Likert scale)		1 (IQR 0)	2 (IQR 1)	

Average values for all readers and additionally *p*-values for each reader in brackets (tumor size, area, and T-stage as measured in the monoenergetic photon-counting detector computed tomography reconstruction and tumor size and area as measured also in the iodine map). Mean values are accompanied by SD, median values by IQR

*IQR* Interquartile range, *NAST* Neoadjuvant systemic therapy, *SD* Standard deviation

### Lymph node metastasis

Regarding axillary lymph nodes, there was a significant decline in the presence and number of visually suspicious lymph nodes (both averaged *p* < 0.001, Table 4). Before NAST visually suspicious axillary lymph nodes were detected by PCCT in 5/3/5 (reader 1/2/3) out of 9 cases. After NAST, visually suspicious axillary lymph nodes were detected by PCCT in 1/0/0 (reader 1/2/3) out of 9 cases. Histopathology of post-NAST surgery revealed 2 residual lymph node metastases in only one case (case 9, ypN1a 2/11), which was not detected by PCCT reading. However, these lymph node metastases only had a maximum size of 3 mm in one lymph node and only single tumor cells in the second lymph node, therefore no major lymph node enlargement or deformity could be expected in the PCCT examination.

These results are also displayed in Supplementary Table S3. An example of a lymph node metastasis in MRI and PCCT before NAST and its decline in PCCT after NAST is shown in Fig. 5.

### Tumor response

Altogether, PCCT correctly identified a decrease in tumor burden as a response to NAST in all cases. Pathological complete response was detected in 2/9 cases. Both cases of pathological complete response were correctly identified by PCCT. In contrast, 7/9 cases only showed partial response in histopathology. Of these 7/9 cases, PCCT identified 5/7 cases correctly as partial response, while histopathological minimal residual tumor burden was underestimated as complete response by PCCT in 2/7 cases (Fig. 2). One of these cases had initially diffuse tumor spread in stage cT4 with a marked decline after NAST to histopathological residual tumor burden in stage yT1 (yT1c with 11 mm, Fig. 2, tumor 8). The second case initially presented in stage cT2 and showed only *in situ* residual disease after NAST in histopathology (ypTis, Fig. 2, tumor 3). One further case of diffuse tumor spread in stage cT4 showed no clearly enhancing tumor areas after NAST in PCCT but minimal residual tumor burden in histopathology (yT1a with 3 mm, Fig. 2, tumor 6).

### Iodine uptake in tumors before and after NAST

In case of depictable residual tumor masses after NAST (5/9), mean and maximum iodine uptake only showed a nonsignificant reduction (average *p* = 0.092 and 0.363). Correspondingly, the reduction in mean tumor density related to mean HU measurements in the aorta was not statistically significant after NAST (average for monoenergetic reconstruction and iodine map: *p* = 0.156 and 0.406). Only when assuming that in case of non-detectable residual tumor after NAST, iodine uptake is “0” in mg/mL, a significant reduction in mean and maximum iodine uptake is observed after NAST (mean iodine uptake: averaged *p* < 0.001, reader 1/2/3 *p* = 0.027/0.027/0.029; maximal iodine uptake: averaged *p* < 0.001, reader 1/2/3 *p* = 0.027/ 0.020/0.019).

### Image quality and certainty of findings

Image quality in PCCT was rated to be very good before and after NAST (median = 1/1/1, IQR 0/0/0 for reader 1/2/3, respectively) without any significant difference (*p* = 1). Certainty of findings was rated to be very good before (median = 1/1/1, IQR 0/0/1 for reader 1/2/3) and good to very good after NAST (median = 2/1/1, IQR 1/1/1 for reader 1/2/3). These results are also summarized in Table 4 with averaged values for all readers.

### Inter-rater reliability

Before NAST inter-rater reliability for main unenhancing tumor size in the monoenergetic reconstruction was 0.97, for main enhancing tumor size and area in the monoenergetic reconstruction 0.89 and 0.97, and in the iodine map 0.88 and 0.98 (each *p* < 0.001) with a good to excellent agreement. Inter-rater agreement for cutis infiltration was perfect (1, *p* < 0.001), for number of suspicious lesions (0.68, *p* < 0.001) moderate and presence of any suspicious axillary lymph nodes (0.70, *p* < 0.001) substantial, for wall infiltration (0.46, *p* = 0.017) and the number of suspicious axillary lymph nodes (0.44, *p* < 0.001) fair. Measurements of mean tumor density resulted in an inter-rater reliability of 0.36 (*p* = 0.039, poor agreement) in the monoenergetic reconstruction, in a moderate agreement of 0.66 (*p* < 0.001) in the iodine map including calculation of mean iodine uptake.

After NAST inter-rater reliability for main unenhancing tumor size in the monoenergetic reconstruction was 0.85, for main enhancing tumor size and area in the monoenergetic reconstruction 0.86 and 0.90 with a good to excellent correlation and in the iodine map 0.75 and 0.71 (each *p* < 0.001) with a good to moderate correlation, for number of suspicious lesions 0.80 (*p* < 0.001) with a good agreement. There were too few cases to calculate inter-rater reliability for cutis infiltration despite complete agreement between all raters of not finding any cutis infiltration. Also, inter-rater reliability for wall infiltration and presence as well as number of suspicious axillary lymph nodes could not be evaluated. Measurements of mean tumor density resulted in an inter-rater reliability of 0.79 (*p* = 0.003) with a good agreement in the monoenergetic reconstruction and of 0.91 (*p* < 0.001) with an excellent agreement in the iodine map including calculation of mean iodine uptake.

There was no significant difference of inter-rater reliability for all values equally measurable before and after NAST (*p* = 0.713), indicating that the variance in evaluation between readers did not differ over time and therefore inter-rater differences as such did not relevantly influence the results before and after NAST.

## Discussion

In this study, we investigated the feasibility of prone-positioned contrast-enhanced thoracic PCCT to assess NAST response in breast cancer. Our findings demonstrated that after NAST, PCCT was able to detect significant reductions in tumor size, tumor area, and T-stage. It successfully identified a decrease in tumor burden in response to NAST in all cases, including correctly identifying complete responses in two cases from our small cohort. Additionally, we observed a significant decrease in the number of suspicious axillary lymph nodes, along with a marked reduction in the presence of additional suspicious lesions in the breast, and cutaneous infiltration.

The reduction in tumor size and burden, particularly pathological complete response with no invasive or *in situ* residuals in breast or lymph nodes [21], is one of the primary goals of NAST [21, 22]. Accurately assessing breast tumors and tumor response to NAST is crucial for optimal planning of surgery and subsequent therapy. Currently, MRI is considered the most accurate method for evaluating NAST response [4–6, 23], alongside other imaging modalities such as ultrasonography, digital mammography, breast tomosynthesis [24], and, less commonly, contrast-enhanced mammography [25, 26] or dedicated breast CT [11]. These modalities differ in their ability to visualize tumor, ranging from measuring tumor diameter to assessing functional tumor volume, and several of these modalities involve not only additional devices and time but also application of additional contrast agent. Moreover, different tumors exhibit diverse response to NAST [6]. Therefore, the American College of Radiology developed a catalogue of Appropriateness Criteria [24, 27].

In our study, we chose to compare tumor size, area, and local T-stage, while also evaluating dermal and thoracic wall infiltration—criteria that are assessable with thoracic PCCT [15, 16]—to compare tumor burden before and after NAST. Thoracic PCCT showed significant reductions in all three main criteria, correctly identifying response to NAST. Additionally, PCCT accurately identified complete tumor response in both cases where it was confirmed by histopathology. In two more cases where PCCT suspected a complete response, histopathology only revealed *in situ* residual disease (ypTis) and in another case residual tumor burden in stage yT1 (yT1c with 11 mm) after initial diffuse tumor spread with high breast density (category D). These discrepancies may result from the difficulty in measuring tumor size when it is significantly reduced or the contrast uptake is lowered after NAST, especially in the presence of high breast density.

Due to the dual source technology of the PCCT device we also investigated iodine contrast uptake in tumors before NAST and after NAST in cases where tumors remained detectable post-treatment. This exploration is particularly relevant, given the well-established association of breast cancer with a robust uptake of intravascular contrast agents in MRI [28], thoracic energy-integrating detector CT [29–34], dedicated cone-beam breast CT [35, 36] and dedicated photon-counting breast CT [37]. In our study, there was only a minor, nonsignificant decline in iodine uptake in residual tumors that remained detectable after NAST (5/9 cases). This could be attributed to the very small sample size or the heterogeneity of tumor subtypes, some of which may retain areas of enhancement despite treatment response with decline in size. Assuming that tumors undetectable after NAST exhibit no iodine uptake, the overall reduction in iodine uptake after NAST became significant compared to overall iodine uptake of tumors before NAST. Further studies are needed to explore the iodine uptake in a larger cohort of breast tumors assessed as a potentially valuable additional feature of PCCT, both in general as well as after NAST. After NAST, it would be particularly relevant for cases of visually residual disease or non-responsive tumors based on size measurements, as it may provide insights into activity, blood supply, and perfusion of residual tumor masses without the need for additional imaging.

For assessing axillary nodal status, ultrasonography is the preferred method both before and after NAST, while MRI, if performed, is mainly acknowledged for initial assessment of clinically node-positive cases [24]. In our study, we compared nodal status in PCCT before and after NAST, as PCCT might be utilized to assess lymph node status across various tumor types and locations.

Despite the promising results, our study is limited by its small sample size and single-center design. Nonetheless, it allowed us to demonstrate that thoracic PCCT shows potential to visualize the goal of NAST—tumor size reduction with partial or better complete response.

Furthermore, the still limited availability of PCCT devices is a current constraint. However, the numerous advantages of PCCT with integrated dual-source technology [38], including high spatial resolution, precise iodine uptake assessment, and a versatile range of diagnostic applications, as evidenced by recent literature [14], suggest that PCCT will inevitably become more accessible in the future. Importantly, thoracic PCCT holds promising potential for breast imaging [15, 16], without requiring additional dedicated breast imaging devices in case of a clinical indication to perform a thoracic CT. In addition, not only standard methods such as tomosynthesis show promising results in the application of radiomics [39, 40]. Conventional pretreatment staging CT reveals a predictive value for individual responses to NAST using radiomics [41]. Further studies utilizing radiomics with thoracic PCCT, maybe in combination with other modalities, could enhance the accuracy and diagnostic utility of this modality, especially since the prognostic impact of pathologic complete response may vary by tumor subtype [21, 22, 42].

Patients in our study only received thoracic PCCT for clinical indications such as thoracoabdominal staging in the first place and follow-up or clarification of complications and suspicious findings after NAST. Therefore, no additional radiation dose or contrast agent was applied for breast diagnostics. Nonetheless, despite the relatively lower radiation dose of PCCT compared to multidetector-row CT, established methods for assessing response to NAST should remain the preferred choice when there are no other clinical indications for thoracic PCCT. Anyway, many breast cancer patients undergo initial thoracoabdominal breast cancer staging, and in this context, PCCT provides a promising possibility to also assess local breast cancer [15, 16]. Afterward, at least some patients may require further thoracic or thoracoabdominal CT examinations to visualize complications or initially unclear findings. For those cases, PCCT might include a promising assessment of changes in local breast disease without further dedicated imaging.

Future research might also explore the use of PCCT in supine positioning, which is more commonly performed in clinical practice.

In conclusion, with the presented initial promising results, our study demonstrates the feasibility of PCCT in accurately assessing the impact of NAST on breast cancer and indicates the need for larger studies. Given the necessity of thoracoabdominal staging CT in many breast cancer patients and in some cases of thoracic or thoracoabdominal follow-up CT examinations, the significant and accurately detected reductions in tumor size following NAST underscore the potential of contrast-enhanced prone-positioned thoracic PCCT as a valuable tool for monitoring treatment response in routine clinical practice in case of appropriate indications for thoracic PCCT.

## Supplementary information

**Additional file 1:** **Supplementary Table 1.** Comparison of tumor size and stage in MRI and PCCT before and in histopathology and PCCT after neoadjuvant systemic therapy. Measurements in PCCT include unenhancing (PCCT unenhanced) and enhancing tumor size (PCCT enhanced) in the monoenergetic 65 keV reconstructions and enhancing tumor size in the iodine map (PCCT iodine map) for reader 1/2/3, respectively. For case 1 and 9 the repeated measurements of total tumor size in PCCT after neoadjuvant systemic therapy performed by reader 1 and 2 are shown in brackets. **Supplementary Table 2.** Number of suspicious lesions, cutis and pectoralis muscle infiltration in PCCT before and after neoadjuvant systemic therapy as assessed by reader 1/2/3. **Supplementary Table 3.** Number of suspicious axillary lymph nodes in PCCT before and after neoadjuvant systemic therapy as assessed by reader 1/2/3 and histopathologically detected number of lymph node metastasis by axilla surgery after neoadjuvant systemic therapy.

## Data Availability

The datasets used and/or analyzed during the current study are available from the corresponding author upon reasonable request.

## Abbreviations

CT: Computed tomography
IQR: Interquartile range
MRI: Magnetic resonance imaging
NAST: Neoadjuvant systemic therapy
PCCT: Photon-counting detector CT
