# Supplementary material for: Breast cancer assessment under neoadjuvant systemic therapy using thoracic photon-counting detector computed tomography in prone position: a pilot study
Source: Eur Radiol Exp. 2025 Mar 28;9:41. doi: 10.1186/s41747-025-00576-z (PMC11953491; doi:10.1186/s41747-025-00576-z)
Supplement: Supplementary file 1 — Additional file 1: Supplementary Table 1. Comparison of tumor size and stage in MRI and PCCT before and in histopathology and PCCT after neoadjuvant systemic therapy. Measurements in PCCT include unenhancing (PCCT unenhanced) and enhancing tumor size (PCCT enhanced) in the monoenergetic 65 keV reconstructions and enhancing tumor size in the iodine map (PCCT iodine map) for reader 1/2/3, respectively. For case 1 and 9 the repeated measurements of total tumor size in PCCT after neoadjuvant systemic therapy performed by reader 1 and 2 are shown in brackets. Supplementary Table 2. Number of suspicious lesions, cutis and pectoralis muscle infiltration in PCCT before and after neoadjuvant systemic therapy as assessed by reader 1/2/3. Supplementary Table 3. Number of suspicious axillary lymph nodes in PCCT before and after neoadjuvant systemic therapy as assessed by reader 1/2/3 and histopathologically detected number of lymph node metastasis by axilla surgery after neoadjuvant systemic therapy. [file 41747_2025_576_MOESM1_ESM.pdf]

Breast cancer assessment under neoadjuvant systemic therapy using thoracic photon-counting detector computed tomography in prone position: a pilot study

ELECTRONIC SUPPLEMENTARY MATERIAL

Supplementary Table 1. Comparison of tumor size and stage in MRI and PCCT before and in histopathology and PCCT after neoadjuvant systemic therapy. Measurements in PCCT include unenhancing (PCCT unenhanced) and enhancing tumor size (PCCT enhanced) in the monoenergetic 65 keV reconstructions and enhancing tumor size in the iodine map (PCCT iodine map) for reader 1/2/3, respectively. For case 1 and 9 the repeated measurements of total tumor size in PCCT after neoadjuvant systemic therapy performed by reader 1 and 2 are shown in brackets.

	Before neoadjuvant systemic therapy						After neoadjuvant systemic therapy					
Case	MRI [mm]	MRI T stage	PCCT unenhanced [mm]	PCCT enhanced [mm]	PCCT iodine map [mm]	PCCT T stage	Histopathology [mm]	ypT	PCCT unenhanced [mm]	PCCT enhanced [mm]	PCCT iodine map [mm]	PCCT T stage
1	16	4	12/10/17	12/10/17	12/10/11	4/4/4	25	2	9/9/9 (23/24)	2/3/4 (23/24)	2/3/3	1/1/1 (2/2/1)
2	21	2	21/20/21	21/20/21	21/20/20	2/1/2	0	0	0/0/0	0/0/0	0/0/0	0/0/0
3	28	2	27/25/27	27/25/27	27/25/27	2/2/2	8	1	0/0/0	0/0/0	0/0/0	0/0/0
4	NA	NA	20/16/18	20/16/18	19/11/14	1/1/1	0	0	0/0/0	0/0/0	0/0/0	0/0/0
5	15	1	14/14/14	14/14/14	12/12/12	1/1/1	2	1	7/7/8	7/7/8	5/5/5	1/1/1
6	31	4	47/45/49	23/25/49	23/22/48	2/4/4	3	1	30/28/33	0/0/0	0/0/0	0/0/4
7	20	1	20/19/29	20/19/26	19/13/18	1/1/2	12	1	10/9/9	10/9/9	7/6/7	1/1/1
8	90	4	74/87/83	60/67/66	60/56/59	4/4/4	11	1	0/0/0	0/0/0	0/0/0	0/0/0
9	32	2	31/31/31	31/31/31	31/32/31	2/2/2	23	2	20/0/0 (23/18)	7/0/0 (23/18)	7/0/0	1/0/0 (2/1/0)

MRI Magnetic resonance imaging, *PCCT* Photon-counting detector computed tomography

Supplementary Table 2. Number of suspicious lesions, cutis and pectoralis muscle infiltration in PCCT before and after neoadjuvant systemic therapy as assessed by reader 1/2/3.

	**Before neoadjuvant systemic therapy**			**After neoadjuvant systemic therapy**		
**Case**	**Number of suspicious lesions [number]**	**Cutis infiltration [yes/no]**	**Pectoralis muscle infiltration [yes/no]**	**Number of suspicious lesions [number]**	**Cutis infiltration [yes/no]**	**Pectoralis muscle infiltration [yes/no]**
1	6/6/3	y/y/y	n/n/n	6/6/1	n/n/n	n/n/n
2	1/1/1	n/n/n	n/n/n	0/0/0	n/n/n	n/n/n
3	6/6/2	n/n/n	n/n/n	0/0/0	n/n/n	n/n/n
4	6/4/5	n/n/n	n/n/n	0/0/0	n/n/n	n/n/n
5	6/6/6	n/n/n	n/n/n	6/6/6	n/n/n	n/n/n
6	1/1/2	n/n/n	n/y/y	2/1/2	n/n/n	n/n/y
7	2/3/3	n/n/n	n/n/n	4/3/3	n/n/n	n/n/n
8	6/6/6	y/y/y	n/n/n	0/0/0	n/n/n	n/n/n
9	3/3/6	n/n/n	n/n/n	2/2/0	n/n/n	n/n/n

y Yes, *n* No

Supplementary Table 3. Number of suspicious axillary lymph nodes in PCCT before and after neoadjuvant systemic therapy as assessed by reader 1/2/3 and histopathologically detected number of lymph node metastasis by axilla surgery after neoadjuvant systemic therapy.

	**Before neoadjuvant systemic therapy**	**After neoadjuvant systemic therapy**	
**Case**	**PCCT lymph nodes [number]**	**Histopathology lymph nodes [number]**	**PCCT lymph nodes [number]**
1	0/0/0	0	0/0/0
2	0/0/0	0	0/0/0
3	0/0/0	0	0/0/0
4	3/0/5	0	0/0/0
5	3/2/1	0	0/0/0
6	1/0/1	0	0/0/0
7	0/0/0	0	0/0/0
8	6/6/6	0	1/0/0
9	1/1/6	2	0/0/0

PCCT Photon-counting detector computed tomography
